# Effects of adductor canal block on pain management compared with epidural analgesia for patients undergoing total knee arthroplasty: A randomized controlled trial protocol

**DOI:** 10.1097/MD.0000000000021672

**Published:** 2020-08-28

**Authors:** Lianzhou Zhu, Li Yang, Zhengkai Wang, Hanjuan Cui

**Affiliations:** a Department of Anesthesiology, Changji Branch of the First Affiliated Hospital of Xinjiang Medical University; b Department of Gynaecology and Obstetrics, Changji First People's Hospital; c Department of Intensive Medicine, Changji Branch of the First Affiliated Hospital of Xinjiang Medical University, Xinjiang, China.

**Keywords:** adductor canal block, epidural analgesia, pain control, protocol, random, total knee arthroplasty

## Abstract

**Background:**

Total knee arthroplasty (TKA) is known to be a painful orthopedic procedure and moderate to severe pain is common, especially immediately postoperatively and during active motion. The aim of the present study was to compare epidural analgesia (EA) and adductor canal block (ACB) techniques with regard to early period pain levels, need for additional opioids, and ambulation and functional scores in patients who had undergone primary TKA.

**Methods:**

Approval for the study was granted by the Changji Branch of the First Affiliated Hospital of Xinjiang Medical University. Written informed consent will be obtained from all of the participants. Inclusion criteria included the following: planned unilateral TKA; spinal anesthesia; American Society of Anesthesiologists physical status classification score of I to III. Prospective assessment will be done for 100 patients who are scheduled for unilateral primary TKA surgery in our academic hospital by a single senior surgeon between August 2020 and December 2021. Patients were randomized to ACB treatment or EA treatment by a computer random number generator. The primary outcome was visual analog scale pain scores in the immediate postoperative period. Secondary outcomes included postoperative opioid use, length of hospital stay, activity level during physical therapy, and knee range of motion. Results were evaluated in a confidence interval of 95% and at a significance level of *P* < .05.

**Conclusions:**

We hypothesized that standard ACB would be as effective as EA for postoperative pain management following TKA.

**Trial registration:**

This study protocol was registered in Research Registry (researchregistry5775).

## 1 Introduction

The prevalence of knee osteoarthritis is increasing world-wide due to the aging population. A standard surgical intervention for advanced knee osteoarthritis is total knee arthroplasty (TKA), and demand for this surgery is rising.^[1]^ TKA is known to be a painful orthopedic procedure and moderate to severe pain is common, especially immediately postoperatively and during active motion.^[2,3]^ The proportion of patients complaining of chronic pain after TKA is as much as 34%, and the intensity of early postoperative pain is associated with increased chronic pain after TKA. Therefore, postoperative pain management is of utmost importance for patient outcome and satisfaction, and many studies have reported that multimodal pain management was necessary.^[4,5]^

Patient-controlled analgesia, local infiltration analgesia, epidural analgesia (EA), femoral nerve block (FNB), and adductor canal block (ACB) are some methods that may manage the postoperative pain and shorten the physiotherapy duration, but the most efficacious remains unclear.^[6,7]^ EA consisting of a local anesthetic agent and an opioid has been a regular regimen used for postoperative analgesia after TKA. However, some studies have indicated that the benefit of EA must be weighed against the frequency of its adverse effects such as urinary retention, hypotension, pruritus, and motor block that delays mobilization.^[4,8–10]^ FNB has traditionally been the gold standard for analgesia following TKA, but FNB significantly impairs quadriceps motor function, which may interfere with rehabilitation and delay discharge. ACB has emerged as an alternative to FNB after TKA. ACB offers the advantage of sparing the motor nerve supply to most of the quadriceps muscle, which may facilitate physiotherapy after TKA and may lead to a reduction in falls after surgery. ACB is commonly integrated into a multimodal pain protocol to improve pain management after TKA.^[11–14]^

However, while studies exist comparing FNB to EA and FNB to ACB,^[8,9,15]^ there have been limited studies directly comparing ACB to EA in terms of postoperative pain control and ambulation after primary TKA.^[16–18]^ Thus, the aim of the present study was to compare EA and ACB techniques with regard to early period pain levels, need for additional opioids, and ambulation and functional scores in patients who had undergone primary TKA. We hypothesized that standard ACB would be as effective as EA for postoperative pain management following TKA.

## 2 Material and method

### 2.1 Study design and patient enrolment

Approval for the study was granted by the Changji Branch of the First Affiliated Hospital of Xinjiang Medical University (CJ97440). Written informed consent will be obtained from all of the participants. Prospective assessment will be done for 100 patients who are scheduled for unilateral primary TKA surgery in our academic hospital by a single senior surgeon between August 2020 and December 2021. Our study was registered in Research Registry (researchregistry5775) prior to the enrollment start. All surgeons, recovery room and floor nurses, research assistants, statisticians, and patients were blinded to group allocation. Only the anesthesiologists performing the blocks and operating room nurses were not blinded.

Inclusion criteria included the following: planned unilateral TKA; spinal anesthesia; American Society of Anesthesiologists physical status classification score of I to III. Exclusion criteria included the following: unwillingness to participate in the study; general anesthesia; contraindications for the application of ACB such as localized infection and neurological disease in the lower extremity; history of epilepsy; arrhythmia; alcohol or drug dependency; known allergy to local anesthetics; insufficient co-operation for the completion of the visual analog scale (VAS) for pain scores; patients who had an inability to communicate verbally or who were unwilling to give informed consent; American Society of Anesthesiologists physical status classification score of IV.

### 2.2 Randomization

**Figure 1:**
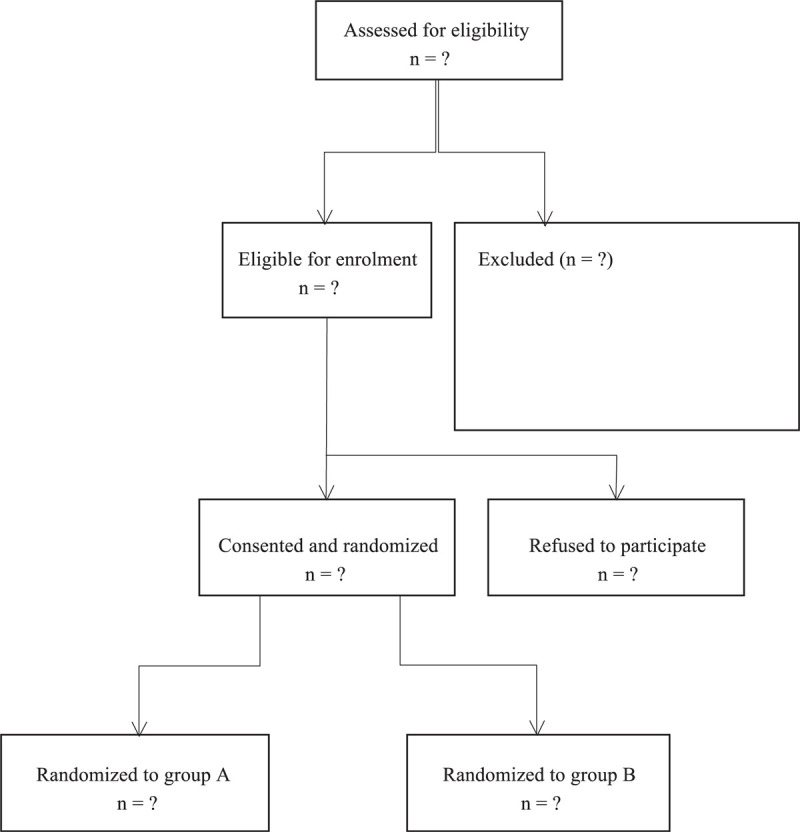
Consolidated standards of reporting trials statement flow diagram.

Patients were randomized to ACB treatment or EA treatment by a computer random number generator. Every participant received a consecutive study number from 1 to 100 and received the treatment assigned according to the randomization list. The randomization key was first broken when all enrolled patients had completed the study (Fig. 1).

### 2.3 Intraoperative interventions

Spinal anesthesia was induced with 3.0 mL 0.5% hyperbaric bupivacaine at the L3/4 interspaces (alternatively at the L2/3 or L4/5 interspaces). Sedation using propofol and intravenous fluid therapy during surgery was administered at the discretion of the anesthetist.

An epidural catheter was placed laterally at the L3/4 or L4/5 level using a 17-gauge Tuohy needle and inserted upward by 5 cm. A test dose of 1% lidocaine (50 mg) was initially injected, and the sensory block was tested after 10 min by applying ice to the ipsilateral thigh. Then, continuous epidural infusion for management of postoperative pain using the catheter that was inserted preoperatively for use during the TKA operation. 20 mL of 0.3% ropivacaine were administered and 0.2% ropivacaine was infused at 5 mL/h for 36 h.

Under ultrasound guidance as above, a 10-cm, 18-gauge Tuohy needle was introduced into the adductor canal. Following dilation of the adductor canal with normal saline, a 21-gauge nerve catheter was threaded up to 3 to 5 cm beyond the needle tip. The guidewire was removed upon the catheter exiting the needle tip while threading to avoid inadvertent advancement of the catheter out of the space. The catheter was then manipulated and normal saline injected to confirm the catheter tip location within the adductor canal on ultrasound visualization, with peri-arterial spread as the endpoint. Up to 5 to 10 mL of normal saline was used in total per catheter placement. 20 mL of 0.3% ropivacaine was then injected via the catheter, following which a continuous infusion of 0.2% ropivacaine commenced at 5 mL/h for 36 h and then removed.

### 2.4 Surgical procedure and perioperative management

All of the operations were performed by a single senior surgeon, using a tourniquet and a medial parapatellar approach. Cruciate-retaining implants were used in all cases, as none of the patients were inflammatory arthritis or required posterior cruciate ligament resection and the patella was not changed in any patient. Intra-articular analgesic infiltration was not applied to any patient. For deep vein thrombosis prophylaxis, 40 mg enoxaparin sodium was applied subcutaneously once a day for 4 weeks after discharge.

Preoperative prophylactic intravenous 1 g cefazolin were administered to all patients and postoperative antibiotics were continued for 24 h. In patients with a known allergy to penicillin, 500 mg vancomycin was preferred for prophylaxis. If serum creatinine levels were normal, a dose of 75 mg diclofenac sodium in 100 cc saline was given 8 hourly, for patient controlled analgesia. Otherwise infusion of 1000 mg paracetamol was administered. In both groups, 50 mg tramadol was given as rescue analgesia at the request of the patient inpresenceof intolerable pain despitethe use of standard analgesic regimen.

### 2.5 Outcomes and measures

The primary outcome was VAS pain scores in the immediate postoperative period (postoperative day 0 through 3). VAS scores were recorded by nursing staff, blinded to treatment group, every 6 h throughout the hospital stay. VAS scores on each postoperative day were averaged, and the daily averages were used for analysis. Secondary outcomes included postoperative opioid use, length of hospital stay, activity level during physical therapy, and knee range of motion. Total opioid consumption was calculated by converting opioids consumed to morphine equivalents. Length of hospital stay was calculated by measuring the time from the completion of surgery through discharge for each patient. Activity level during physical therapy was recorded by measuring the steps taken in daily physical therapy sessions. Knee range of motion was measured by the surgeon using a goniometer in the office at 3 weeks postoperatively.

### 2.6 Sample size calculation

The sample size calculation was based on a pilot study that we conducted on 20 patients. In this prior study, the mean difference and standard deviation of the VAS scores on postoperative day 0 between the ACB and EA groups were 0.42 and 0.21, respectively. From this, it was determined that 50 subjects would be required to reach an α value of 0.05 and a power of 90%. It was estimated that the attrition rate due to canceled surgery or reasons of late patient ineligibility could be up to 20% and, therefore, to account for this, the final sample size selected was n = 120 (60 per group).

### 2.7 Statistical analysis

Statistical analyses were conducted using SPSS v22.0 software (IBM, Chicago, IL). Conformity of the data to normal distribution was tested with the Kolmogorove–Smirnov test. Independent two samples *t* test was used for comparison of continuous variables and Pearson Chi Square test was used for comparison of categorical variables. Results were evaluated in a confidence interval of 95% and at a significance level of *P* < .05.

## 3 Discussion

Patients undergoing TKA have moderate to severe pain postoperatively. Among the techniques developed for analgesia, the use of regional anesthesia techniques such as FNB has proven efficacious. The drawback of FNB is that they tend to result in motor blockade of the quadriceps muscle and potentially delay postoperative mobilization, as well as increase the risk of falls.^[3–5]^ ACB has been demonstrated to be an effective alternative to the FNB, providing similar analgesic efficacy while sparing the motor strength significantly. However, there have been no studies directly comparing ACB to lumbar EA following TKA. Thus, the aim of the present study was to compare EA and ACB techniques with regard to early period pain levels, need for additional opioids, and ambulation and functional scores in patients who had undergone primary TKA. We hypothesized that standard ACB would be as effective as EA for postoperative pain management following TKA.

## Author contributions

**Conceptualization**: Lianzhou Zhu.

**Data curation**: Li Yang.

**Formal analysis**: Lianzhou Zhu, Li Yang.

**Funding acquisition**: Lianzhou Zhu.

**Investigation**: Lianzhou Zhu, Li Yang.

**Methodology**: Hanjuan Cui.

**Resources**: Zhengkai Wang.

**Software**: Zhengkai Wang.

**Supervision**: Zhengkai Wang.

**Validation**: Hanjuan Cui.

**Visualization**: Zhengkai Wang.

**Writing** – **original draft**: Lianzhou Zhu, Li Yang.

**Writing** – **review & editing**: Zhengkai Wang.
